# Impact of geometric distortion on dose deviation for photon and proton treatment plans

**DOI:** 10.1002/acm2.13517

**Published:** 2022-02-02

**Authors:** Yue Yan, Jinzhong Yang, Yuting Li, Yao Ding, Mo Kadbi, Jihong Wang

**Affiliations:** ^1^ Department of Radiation Oncology St. Jude Children's Research Hospital Memphis Tennessee USA; ^2^ Department of Radiation Physics The University of Texas MD Anderson Cancer Center Houston Texas USA; ^3^ MR Therapy Philips Healthcare Houston Texas USA

**Keywords:** geometric distortion, MRI guided radiation therapy, proton therapy

## Abstract

We investigated the dose deviation related to geometric distortion and dose gradient on magnetic resonance‐only treatment planning for intensity‐modulated radiation therapy and proton therapy. The residual geometric distortion of two different magnetic resonance imaging (MRI) sequences (**A**) and (**B**) was applied in the computed tomography image and the structure set of each patient through a polynomial MRI geometric distortion model to simulate MRI‐based treatment planning. A 3D histogram was generated to specify the relationship of dose deviation to geometric distortion and dose gradient. When the dose gradient (G_d_) approached zero, the maximum dose deviation reached 1.64% and 2.71% for photon plans of sequences **A** and **B**, respectively. For proton plans, the maximum dose deviation reached 3.15% and 4.89% for sequences **A** and **B**, respectively. When the geometric distortion (*d*) was close to zero, the maximum dose deviation was less than 0.8% for photon and proton plans of both sequences. Under extreme conditions (*d* = 2 mm and G_d_ = 4.5%/mm), the median value of dose deviation reached 3% and 3.49% for photon and proton plans, respectively for sequence **A**, and 2.93% and 4.55% for photon and proton plans, respectively, for sequence **B**. We demonstrate that the dose deviation is specific to MRI hardware parameters. Compared to the photon plan, the proton plan is more sensitive to the changes in geometric distortion. For typical clinical MRI geometric distortion (*d* ≤2 mm), the median dose deviation is expected to be within 3% and 5% for photon and proton plans, respectively.

## 1 INTRODUCTION

In recent years, there has been an increasing interest in the application of magnetic resonance imaging (MRI) in radiation therapy.^1^, ^2^, ^3^, ^4^ MRI offers better soft‐tissue contrast compared to traditional computed tomography (CT) without giving extra radiation to the patient. This is critically important because of the escalated dose to the target and surrounding organs at risk (OARs). MRI is also a versatile imaging modality for functional imaging that can be used to evaluate treatment outcomes.^5^, ^6^ MRI‐guided linear accelerator (linac) systems such as ViewRay® MRIdain and Elekta Unity systems are becoming increasingly popular in radiation therapy.^7^, ^8^ These cutting‐edge adaptive radiation therapy systems use MRI daily and can redefine standard clinical practices and ensure the quality of patient care in radiation oncology.

CT has been used as a standard approach for dose calculation, target delineation, and verifying daily setups. MRI is usually co‐registered with CT for soft tissue visualization. However, this introduces a registration uncertainty that compromises the accuracy of dose delivery. Studies have estimated that the magnitude of such errors can be up to 3.5 mm.^9^, ^10^, ^11^ The MRI‐only radiation therapy can eliminate such errors and reduce the unnecessary dose from CT, which is especially beneficial for pediatric patients. The MRI‐only planning is increasingly appealing to these patients, owing to the rapid development of MRI‐guided radiation therapy techniques.

A challenge when using MRI‐only radiation therapy is the non‐unique relationship between the signal density and electron density. Due to the complexity of the imaging mechanism, the MRI signal intensity is determined by many factors such as proton density, tissue relaxation properties, and strength of the primary magnetic field. Additionally, the intrinsic geometric distortion of MRI could also compromise the accuracy of target delineation and dose delivery.^12^, ^13^

Several techniques have been developed to introduce an MRI‐only radiation therapy workflow.^14^, ^15^, ^16^ However, the intrinsic geometric distortion cannot be eliminated. In this study, the imaging distortion refers to the residual geometric distortion that is the remaining distortion after implementing the MRI distortion correction algorithm provided by the vendor. Several studies have evaluated the magnitude of geometric distortion in MRI^17^ and its dosimetric impact on photon‐based radiation therapy.^4^, ^18^ However, all these studies were based on specific clinical cases and the relationship of geometric distortion to dose deviation remains unclear. In this study, we investigate the relationship of dose deviation in photon and proton treatment plans with the geometric distortion and dose gradient for the T1‐weighted incoherent gradient echo sequence and the fast field echo sequence of the 1.5 T Elekta Unity system.

## 2 METHODS

### 2.1 Patient selection

The study cohort included 20 anonymous patients selected from the database of the Department of Radiation Oncology at St. Jude Children's Research Hospital (SJCRH) who received intensity‐modulated radiation therapy (IMRT) or proton treatment between 2018 and 2019. This study was reviewed and approved by the St. Jude institutional review board. The age range of patients was 19 months to 21 years. We collected DICOM files of patient CT, structure set, dose prescription, beam angle, and optimization objects. For photon and proton treatments, 10 patients were in each treatment modality group. Target sites for therapy included the brain, prostate, pelvis, spine, abdomen, and extremities. We also used a small number of patients in each study group for multiple cancer sites to cover a broad spectrum of clinical treatment scenarios.

### 2.2 Simulating dose deviation caused by MRI distortion

In this study, we focused on the imaging component (Marlin, Philips Healthcare, Amsterdam, Netherlands) of the 1.5 T Unity (Elekta, Stockholm, Sweden) MRI‐linac system. Two MRI sequences that are widely used in clinical MRI acquisition were used in this study. For sequence **A**, vendor‐provided 3D geometric distortion data for a T1‐weighted incoherent gradient echo sequence were used. Data were acquired by a geometric quality assurance (QA) phantom with 1932 oil markers in 3D space. The diameter of the cylindrical phantom was 50 cm and the length was 33 cm. The interspace among different markers was 25 mm in *x* and *y* directions and 55 mm in the *z*‐direction. For sequence **B**, a 3D T1‐weighted fast field echo sequence was used to scan a 3D geometric QA phantom (Philips) with seven flat plates holding a total of 1932 oil capsules in 3D space. The marker spacing was 25 mm in all three directions, with a 50 cm phantom diameter and a 33 cm length. Sequence parameters of the two sequences are summarized in Table 1.

**TABLE 1:** Parameters of two sequences used in the study

	Sequence **A**	Sequence **B**
Field of View (mm)	560×560×200	560×560×400
Voxel (mm)	2×2×2	1.1×1.1×1.1
Echo Time/Repetition Time (ms)	4.6/11	6.7/3.4
Flip Angle (degree)	30	11
Bandwidth (Hz/pixel)	433	431
Scan Time (min)	3.22	10

All 3D geometric distortion data were fitted into a second‐order polynomial model developed by Yan et al^4^ in each slice to find the optimal free parameters. 3D interpolation was used to simulate the geometric distortion among different slices. Based on the work of Yan et al's,^4^ the simulated error was within 0.4 mm. One difficulty of the study lies in the skewness of the distortion data. Most of the voxel in the volume of interest had relatively small geometric distortion (<1 mm), leading to unstable statistical results in the high geometric distortion region. Data augmentation was used to overcome this challenge. In this study, the magnitude of geometric distortion was manually changed to one time, two times, and three times. The corresponding dose deviation compared to the clinical plan was calculated for each voxel. The 3D histogram statistics consisted of all of the clinical data and the artificially created data.

For patients receiving proton therapy, single‐field uniform dose and intensity‐modulated proton therapy were used to develop the plans. For all patients, target and OARs^19^ associated with the clinical CT had been reviewed and approved by attending radiation oncologists. For photon IMRT plans, the dose was prescribed to the planning target volume. For proton plans, the dose was prescribed to the clinical target volume.^17^ Siemens (Concord, California) Artiste™ linear accelerator (linac) and Hitachi PROBEAT‐V proton systems (Hitachi, Ltd, Hitachi City, Japan) were commissioned on the Varian (Palo Alto, California) Eclipse™ treatment planning system to develop photon and proton plans, respectively. Anisotropic Analytical Algorithm^20^, ^21^, ^22^ (v13.7.14) and Proton Convolution Superposition^23^, ^24^, ^25^ (v15.6.05) were used to calculate photon and proton dose, respectively.

The clinical plan was used as a reference for each patient. Geometric distortion was applied to the clinical CT and the structure set for all patients. New plans were re‐optimized using the same parameters such as gantry angle, beam energy, treatment isocenter, and dose constraints as the reference plan based on the distorted CT and the structure set. The dose of the distorted plan was recalculated by using the clinical CT and the structure set. In this study, we refer to these plans as distorted plans. The final dose deviation was calculated as the difference between the reference dose and the distorted dose. The dose grid was 2.5 mm in all three directions for both photon and proton plans. Dose gradient was calculated as the dose derivative of distance in the reference plan to describe how fast the local dose changes with distance. Dose deviation and dose gradient were calculated for each voxel. The 3D histogram of dose deviation (∆*D*) was generated against geometric distortion (*d*) and dose gradient (*G*_d_).

## 3 RESULTS

**FIGURE 1:**
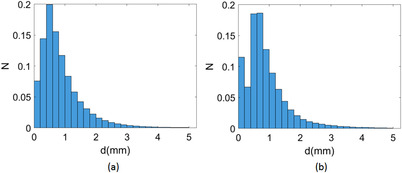
(color online) Normalized geometric distortion histogram of the 1.5 T Unity system for sequence **A** (a) and sequence **B** (b). d represents the magnitude of geometric distortion, N represents the number of data points in each bin of *d*

**FIGURE 2:**
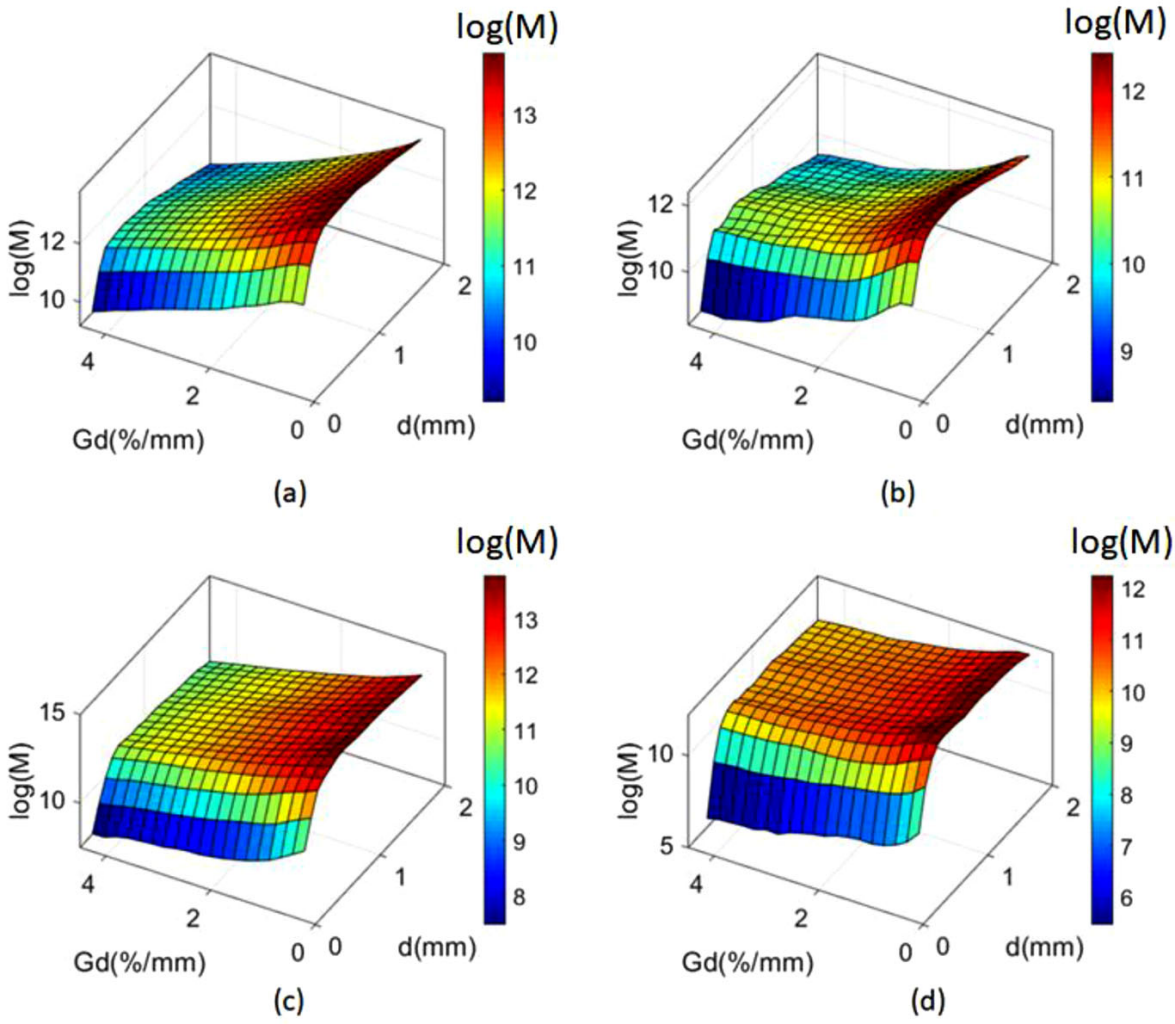
(color online) Number of data points for each statistical bin of sequence **A** (a,b) and sequence **B** (c,d). Data points for photon and proton plans are shown in (a, c) and (b, d), respectively. *d*, *G*_d_, and M are the magnitude of geometric distortion, the dose gradient, and the number of data points at each statistical bin, respectively

The normalized distribution of residual geometric distortion for the Unity system is shown in Figure 1. As seen clearly, most of the distortion was smaller than 1 mm, with a mean value of 0.9 mm for both sequences. A 2D mesh grid was generated by setting the coordinates of geometric distortion (*d*) and dose gradient (*G*_d_). A 3D histogram was created to evaluate the relationship of median dose deviation (∆*D*) to geometric distortion and dose gradient. The natural logarithm of the number of data points (M) in each pixel of the mesh grid is shown in Figure 2. Clearly, with an increase in *d* and *G*_d_, the M gradually decreased for all plans of both sequences. This effect can be understood by the fact that most data points lie within a volume with *d* ≤1 mm, as indicated by Figure 1.

**FIGURE 3:**
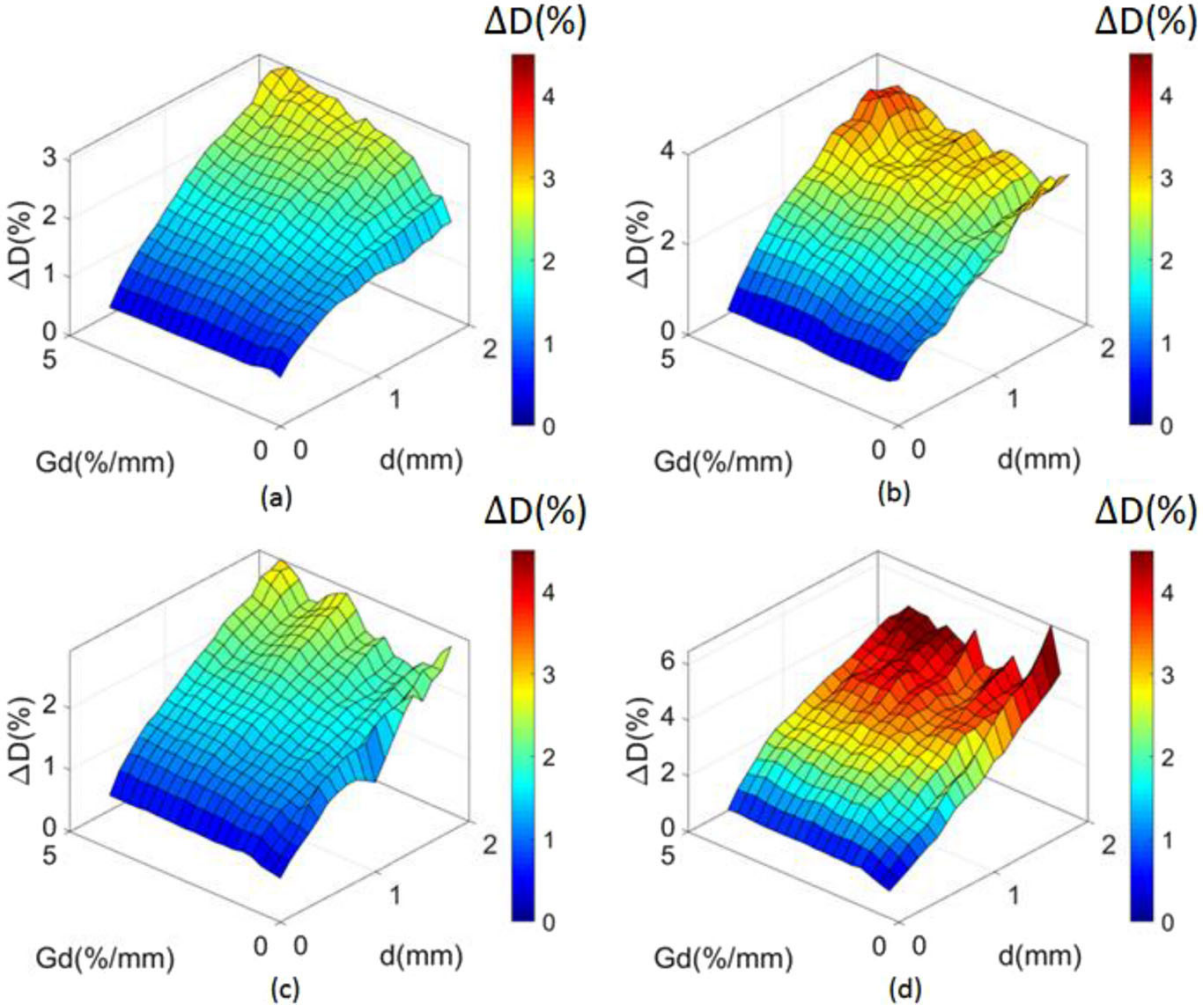
(color online) Dose deviation of sequence **A** (a,b) and sequence **B** (c,d). Photon plan deviations are shown in (a) and (c), proton plan deviations are shown in (b) and (d). *d*, *G*_d_, and Δ*D* are the magnitude of geometric distortion, dose gradient, and the dose deviation, respectively

The 3D histogram of median normalized dose deviation against geometric distortion and dose gradient is shown in Figure 3. As shown, for both sequences, proton plans were more sensitive to changes in geometric distortion and dose gradient. For all results shown in Figure 3, *d* and *G*_d_ were in the range that *d* ≤2 mm and *G*_d_ ≤ 4.5 mm. For sequence **A**, ∆*D*_max_ values were 3.1% and 3.87% for photon and proton plans, respectively. For sequence **B**, ∆D_max_ values were 3.93% and 6.48% for photon and proton plans, respectively. When geometric distortion and dose gradient approached the maximum value (*d* = 2 mm and *G*_d_ = 4.5%/mm), the median of ∆*D* reached 3% and 3.49% for photon and proton plans, respectively, for sequence **A** and 2.93% and 4.55% for photon and proton plans, respectively, for sequence **B**. As shown in Figure 3, dose deviations were related to hardware and sequence parameters, which further demonstrates the complexity of the impact of geometric distortion on dose deviation for photon and proton plans.

**FIGURE 4:**
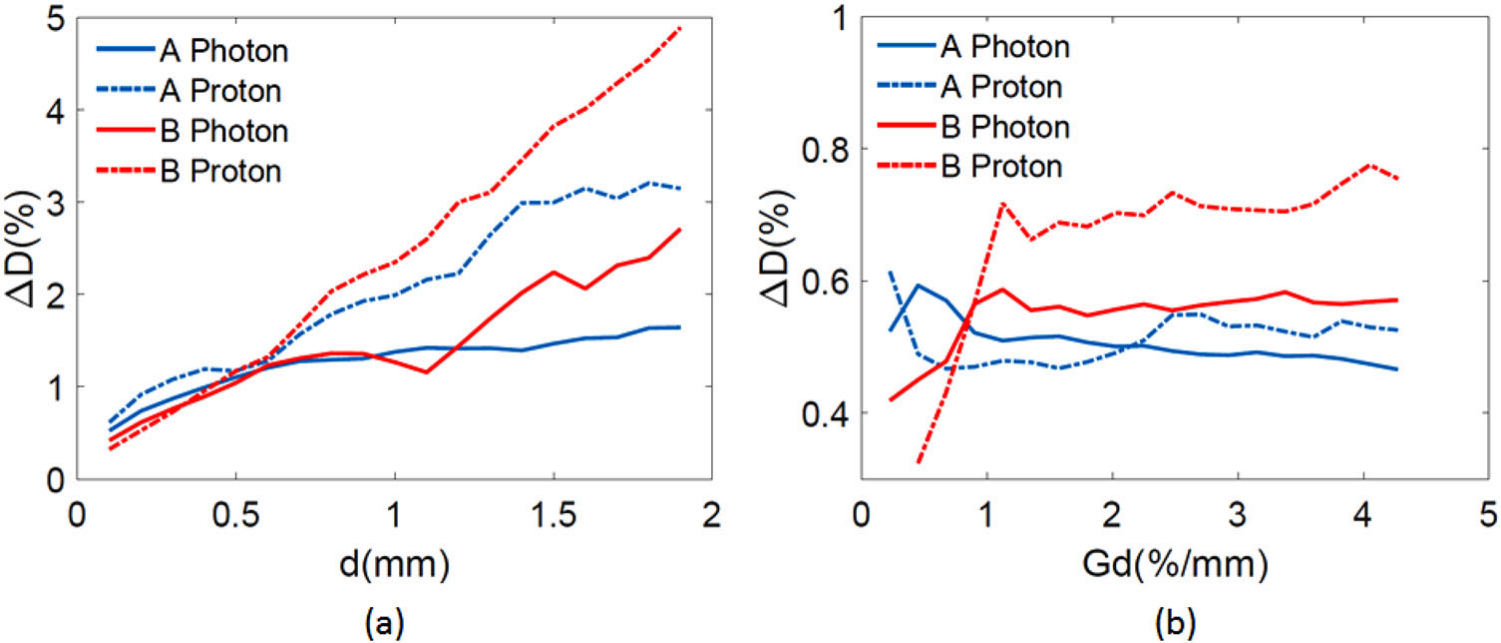
(color online) Dose deviations ΔD at the edge of Figure 3 of sequence **A** and **B** for photon and proton plans. Panel (a) shows the change of Δ*D* with zero dose gradient *G*_d_. Panel (b) shows the change of Δ*D* with zero geometric distortion *d*

Dose deviations with the zero dose gradient and geometric distortion are shown in Figure 4a,b, respectively. Compared to the dose gradient, the geometric distortion was the dominant factor that decided the dose deviations in photon and proton plans. Based on Figure 4a, proton plans were more sensitive to changes in geometric distortion. Overall, dose deviation tended to increase with an increase in geometric distortion. When geometric distortion was close to zero, the increase in dose gradient did not lead to apparent changes in dose deviations in photon and proton plans for both sequences. Specifically, when the dose gradient approached zero, the maximum dose deviation reached 1.64% and 2.71% for photon plans of sequences **A** and **B**, respectively. For proton plans, the maximum dose deviation reached 3.15% and 4.89% for sequences **A** and **B**, respectively. When geometric distortion was close to zero, the dose deviation caused by the increased dose gradient was less than 0.8% for photon and proton plans of both sequences.

## 4 DISCUSSION

In this study, we developed a generalized technique to study the relationship of dose deviations to geometric distortion and dose gradient. Two MRI sequences were used to simulate the dose deviations caused by geometric distortion. A cohort of 20 patients receiving photon and proton treatment with tumors at six different anatomic sites comprised this study group. A polynomial model^4^ was used to simulate the geometric distortion of two MRI sequences. For each voxel, dose deviation, dose gradient of the clinical plan, and the corresponding geometric distortion were calculated. Rather than focusing on a specific cancer category, we used a small number of patients in each study group for multiple cancer sites to cover a broad spectrum of clinical treatments. We also extended our study to state‐of‐the‐art proton therapy. To the best of our knowledge, this is the first study that analyzes the impact of MRI distortion on proton treatment.

In our current study, the uncertainty of re‐optimization in the distorted plan is expected to contribute to the overall dose deviations from the clinical plans. Based on the calculation results shown in Figures 3 and 4, under the condition that d approached zero, median dose deviations caused by the optimization uncertainty were less than 1% of the total dose for all plans. Thus, the minor uncertainties of there‐optimization did not compromise the integrity of our study.

It is known that dose gradient shows how fast the dose change with respect to the change in distance. When the dose gradient is very high (e.g., 5%/mm), small geometric distortion (e.g., <1 mm) could potentially lead to a dose deviation making a plan is unacceptable. The dose gradient is a characteristic of the treatment plan itself, which is decided by planning goals, treatment modality, and hardware parameters such as the MLC leaf width and beam quality. Geometric distortion could lead to more prominent dose deviations in regions with large dose gradients on MR‐based treatment plans, as shown in Figure 4a,b.

It is worth mentioning that our study accounts for only the systematic geometric distortion after standard correction. The geometric distortion data measured by the phantom during our routine QA shows that slightly larger residual distortion can be observed in the peripheral regions, usually with 2 mm at about 20 cm away from the imaging center, compared with negligible distortion within 5 cm of the imaging center.^4^ By applying the geometric distortion model derived from phantom data to patient plans, it will help to guide patient setup and treatment planning for MR‐linac treatment. For example, one may set up the patient so that the treatment target falls within a region with negligible residual systematic distortion. One may also use this study to guide the margin creation to account for geometric distortion, particularly in high‐dose gradient regions. One limitation is that the patient‐specific residual distortion was not considered in this study. Additional study to measure the patient‐specific residual distortion is needed but our approach in analyzing the dose deviation related to this type of residual distortion and dose gradient is still applicable.

The presented study provides a technique to generate an atlas that correlates dosimetric deviations with dose gradient and geometric distortion. Due to the complexity of the MRI geometric distortion, the atlas could be generated for different sequences of a specific MRI‐linac system. The results can be used to predict dose deviations of the treatment plan, which could be used to evaluate the robustness of the MRI‐based treatment plan for photon and proton treatment.

## 5 CONCLUSION

In this study, we developed a technique to correlate the dose deviation with dose gradient and MRI geometric distortion for MRI systems. We demonstrate that the dose deviation caused by the geometric distortion of MRI depends on the specific MRI hardware parameters. Compared to the photon plan, the proton plan is more sensitive to changes in geometric distortion. For typical clinical MRI geometric distortion (*d* ≤2 mm), the median dose deviation is expected to be within 3% and 5% for photon and proton plans, respectively. To fully use the benefits of MRI in radiation therapy, the MRI geometric distortion needs to be carefully addressed for MRI‐based treatment planning.

## AUTHOR CONTRIBUTIONS

The above authors have contributed substantially to the study design, data acquisition and analysis, and interpretation of this work. All authors have reviewed, approved, and agreed to be accountable for all aspects related to the accuracy integrity of this manuscript submission.

## CONFLICT OF INTEREST

The authors have declared no conflict of interest.
